# Systematic analysis reveals cis and trans determinants affecting C-to-U RNA editing in *Arabidopsis thaliana*

**DOI:** 10.1186/s12863-020-00907-6

**Published:** 2020-09-03

**Authors:** Duan Chu, Lai Wei

**Affiliations:** College of Life Sciences, Beijing Normal University, No. 19 Xinjiekouwai Street, Haidian District, Beijing, China

**Keywords:** C-to-U RNA editing, Cis, Trans, Regulatory, *Arabidopsis*, **Synonyms**“Missense” and “nonsynonymous”.

## Abstract

**Background:**

C-to-U RNA editing is prevalent in the mitochondrial and chloroplast genes in plants. The biological functions of a fraction of C-to-U editing sites are continuously discovered by case studies. However, at genome-wide level, the cis and trans determinants affecting the occurrence or editing levels of these C-to-U events are relatively less studied. What is known is that the PPR (pentatricopeptide repeat) proteins are the main trans-regulatory elements responsible for the C-to-U conversion, but other determinants especially the cis-regulatory elements remain largely uninvestigated.

**Results:**

By analyzing the transcriptome and translatome data in *Arabidopsis thaliana* roots and shoots, combined with RNA-seq data from hybrids of *Arabidopsis thaliana* and *Arabidopsis lyrata*, we perform genome-wide investigation on the cis elements and trans-regulatory elements that potentially affect C-to-U editing events. An upstream guanosine or double-stranded RNA (dsRNA) regions are unfavorable for editing events. Meanwhile, many genes including the transcription factors may indirectly play regulatory roles in trans.

**Conclusions:**

The 5-prime thymidine facilitates editing and dsRNA structures prevent editing in cis. Many transcription factors affect editing in trans. Although the detailed molecular mechanisms underlying the cis and trans regulation remain to be experimentally verified, our findings provide novel aspects in studying the botanical C-to-U RNA editing events.

## Key message

In *Arabidopsis thaliana*, the 5-prime nucleotide and the RNA secondary structures affect C-to-U RNA editing in cis while many transcription factors play regulatory roles in trans.

## Background

In the plant kingdom, C-to-U RNA editing is one of the most prevalent RNA modifications and is enriched in the chloroplast and mitochondrial genes [1–6]. The biological functions of particular C-to-U editing events are discovered [7–10]. For example, phenotypic studies have associated mitochondrial C-to-U editing with seed development in maize (*Zea mays*) and rice (*Oryza sativa*) [11–13]. Mechanistic study found that a particular C-to-U editing site in *Oenothera* gene *nad1* is required for proper splicing of pre-mRNA [14]. Early studies in sugar beet (*Beta vulgaris*) and tobacco (*Nicotiana tabacum*) found that mitochondrial C-to-U editing events were able to create translation start codons and produce functionally important proteins [15–17].

Apart from these case studies on editing function, the large-scale identification of C-to-U editing sites appeared in recent years with the development of next generation sequencing (NGS) technique. Bioinformatic tools are published to systematically identify bona fide C-to-U editing sites [18–21] and databases are built to collect the editing sites reported by different researchers [20, 22].

Despite these fruitful findings in functional RNA editing and the convenience for accessing the editing sites, it remains uninvestigated that, at genome-wide level, what are the cis and trans determinants affecting the occurrence or editing levels of these C-to-U events? The established knowledge is that multiple factors are responsible for C-to-U RNA editing in plants, the best-studied of which is the PPR (pentatricopeptide repeat) protein [23]. However, other determinants especially the cis-regulatory elements remain largely undiscovered.

This issue could be partially resolved with the aid of next generation sequencing technique. RNA-seq (or mRNA-seq) typically sequences the fragmented reads from cellular mRNAs and could provide information on the abundance of each transcript. Ribosome profiling followed by deep sequencing [24] captures the ribosome protected mRNA fragments (about 30 nt), allowing a more accurate estimation of gene expression at the translatome level [25]. Thus, the cis features affecting editing sites might be parsed from the mRNA-seq data while the trans acting genes could be inferred from the genome-wide expression profile calculated from ribosome profiling data.

In this study, by analyzing the transcriptome (RNA-seq) and translatome (ribosome profiling) data in *Arabidopsis thaliana* roots and shoots [26, 27], combined with RNA-seq data from hybrids of *Arabidopsis thaliana* and *Arabidopsis lyrata* [28], we perform genome-wide investigation on the cis elements and trans-regulatory elements that potentially affect C-to-U editing events. We find that the 5-prime nucleotide and the RNA secondary structures affect C-to-U RNA editing in cis, and transcription factors might affect editing in trans. Our findings provide novel aspects in studying the botanical C-to-U RNA editing events and should be appealing to the broad phytologists as well as RNA biologists.

## Results

### Identification of bona fide C-to-U RNA editing sites

**Fig. 1:**
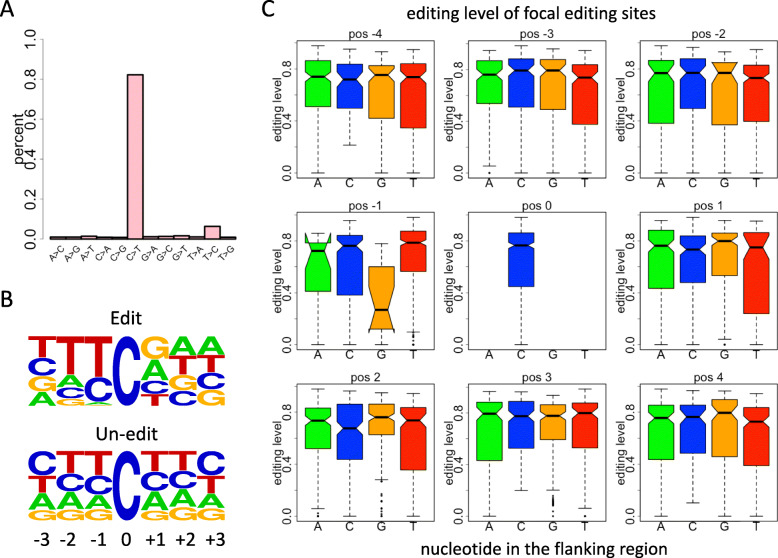
Nucleotide context flanking the C-to-U editing sites. **a** Barplot showing the percentages of different types of variations. **b** Motifs showing the nucleotide context around edited or unedited cytidines. **c** Box-and-whiskers displaying the editing levels of focal editing sites. The focal editing sites are classified into four groups based on the flanking nucleotides from position − 4 to position + 4

Following the results of our recent study [29], using the mRNA-seq from roots and shoots of *Arabidopsis thaliana*, we identify 130 C-to-U RNA editing sites genome-wide, with 12 sites in chloroplast genome, 111 sites in mitochondrial genome, and 7 sites in nucleus genome (3 sites in chromosome 2, 1 site in chromosome 3, and 3 sites in chromosome 4). To demonstrate the reliability of the editing sites we find, we show that the C-to-U alterations compose more than 80% of the total variations detected in mRNA-seq (Fig. 1a) and most of which take place in mitochondrial or chloroplast genes. One hundred thirteen of the 123 bona fide editing sites are detectable in all six mRNA-seq samples (three root samples and three shoot samples). The C-to-U editing sites have remarkably different context compared to the unedited cytidines in the genome (Fig. 1b). Most of the 5-prime nucleotides of edited cytidines are thymidine or cytidine and only a small fraction of upstream nucleotide is guanosine or adenosine, while the context of unedited cytidines is similar to the background nucleotide component (Fig. 1b).

### The 5-prime nucleotide of edited cytidines has great impact on editing levels

The appearance of C-to-U editing sites is context dependent as we have shown (Fig. 1b). However, it is unclear whether the nucleotide context could influence the editing level. We investigate the relationship between editing level and the nucleotide near the editing site (Fig. 1c). Among the eight positions from position − 4 to position + 4 (except position 0 itself), only the nucleotide at position − 1 (the 5-prime nucleotide) has impact on the editing levels of focal cytidines (Fig. 1c). Editing sites with an upstream thymidine have the highest levels and the sites with an upstream guanosine have the lowest levels (Fig. 1c). This pattern might not be surprising because if a 5-prime guanosine is unfavorable for the focal cytidine to be edited, then the editing level (Fig. 1c) should be lower for the cytidines with a 5-prime guanosine.

### Transcriptome-wide analysis combined with hybrids of *A. thaliana* × *A. lyrata* reveal the effect of RNA structure on editing events

**Fig. 2:**
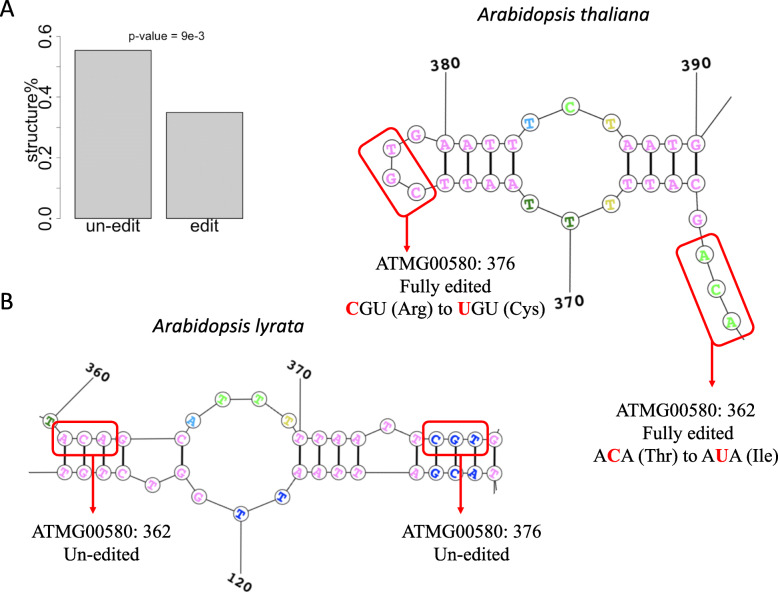
RNA secondary structures affecting the C-to-U editing sites. **a** Barplot showing the percentages of sites located in structured RNA regions. Structured means the hairpin regions defined by software RNAfold. *P*-value is calculated by Fisher’s exact test. **b** RNA structures around two cytidines (position 362 and position 376) on gene ATMG00580 (NADH dehydrogenase subunit 4). The two cytidines are fully edited in *A. thaliana* but completely unedited in *A. lyrata*. C-to-U event at site ATMG00580:362 causes a missense change from ACA (Thr) to AUA (Ile) and C-to-U at site ATMG00580:376 causes a missense change from CGU (Arg) to UGU (Cys)

Apart from the nucleotide context, other cis elements like RNA secondary structures might potentially affect C-to-U editing events. To test this, we employ RNALfold software to determine all the structured RNA regions within each CDS (Materials and methods). Expectedly, edited positions have significantly lower fractions in structured regions compared to the unedited positions (Fig. 2a). Together with the knowledge that the editing factors PPR (pentatricopeptide repeat) proteins are likely to bind single-stranded RNAs [23], it is reasonable that the structured RNA regions are less likely to be edited.

We seek for data to verify our hypothesis. Hybrid individuals are perfect systems to study the effect of cis elements on C-to-U editing events because the two different parental alleles are subjected to identical trans environment. We find mRNA-seq data conducted in the hybrids of *A. thaliana* and *A. lyrata* [30]. With this set of data, we would only focus on the C-to-U editing sites detected in chloroplast and mitochondrial genes. We combine the chloroplast and mitochondrial genomes of these two species and map the hybrid mRNA-seq data to the combined genome (Materials and methods). We extract uniquely mapped reads so that only the regions that are non-identical between *A. thaliana* and *A. lyrata* could be covered (otherwise the read would be mapped at least twice).

Among the 123 C-to-U editing sites we previously identified, we verify two species-specific editing sites with adequate sequencing depth (Materials and methods). Two cytidines, position 362 and position 376 on gene ATMG00580 (NADH dehydrogenase subunit 4), are fully edited in *A. thaliana* (103 reads covered) but completely unedited in *A. lyrata* (31 reads covered). Interestingly, the two cytidines are located in single-stranded RNA regions in *A. thaliana* but in double-stranded regions in *A. lyrata* (Fig. 2b). This finding supports our assumption that C-to-U editing events are favored in single-stranded RNA structures. Moreover, C-to-U event at site ATMG00580:362 causes a missense change from ACA (Thr) to AUA (Ile) and C-to-U at site ATMG00580:376 causes a missense change from CGU (Arg) to UGU (Cys) (Fig. 2b). Thus, the different editing status between *A. thaliana* and *A. lyrata* might lead to the different protein products or functions of gene ATMG00580. Furthermore, it is possible that the two sites reflect the effect of mating i.e. the imprinting effect. However, this is out of the topic of this article and could be investigated in the future.

### Levels of different editing sites are correlated with expressions of different gene sets

We have shown that the cis-elements like the flanking nucleotides and the RNA secondary structures could play a role in determine the occurrence or levels of C-to-U editing sites. Next, we wonder whether we could find any trans regulatory factors affecting the editing levels. Although it is known that PPR proteins are responsible for C-to-U editing events, it does not exclude the possibility that other regulatory factors may also affect the editing levels. A previous study on mammalian A-to-I RNA editing [31] used GTEx (Genotype-Tissue Expression) mRNA-seq data and performed correlation tests between gene expressions and global editing levels, and successfully found (1) a new trans-regulatory elements AIMP2 that might affect the editing process and that (2) ADAR1 and ADAR2 positively contribute to global editing while ADAR3 plays an inhibitory role [31].

**Fig. 3:**
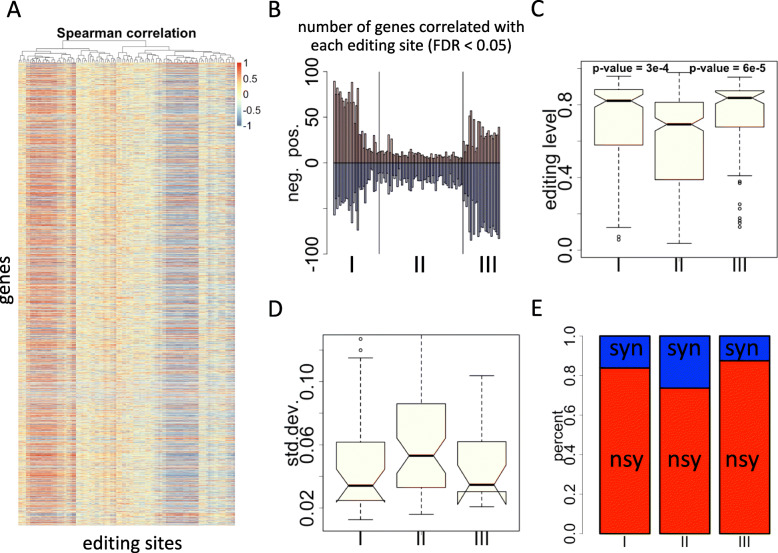
Pairwise correlation between editing levels and gene expression levels (Ribo-seq RPKM) in six samples used in this study. **a** Heatmap showing the Spearman correlation coefficient between editing levels and gene expression levels. **b** Numbers of significantly correlated (multiple testing corrected FDR < 0.05) genes of each C-to-U editing site. The editing sites are classified into three classes based on the numbers of positively and negatively correlated genes. **c** Box-and-whiskers showing the editing levels of three classes of editing sites. *P*-values are calculated by Wilcoxon rank sum tests. **d** Box-and-whiskers showing the standard deviation of editing levels of three classes of editing sites. **e** Barplot showing the fractions of nonsynonymous editing sites among three classes of sites

Enlightened by this study, it is conceivable that if we intend to estimate the protein level of each gene with NGS data, Ribo-seq (ribosome profiling followed by sequencing) should have stronger power than mRNA-seq. With the six mRNA-seq samples and the matched Ribo-seq data, we perform pairwise correlation test between the editing levels of each site in six samples and the expression level (Ribo-seq) of each gene in six samples (Fig. 3a). We use the 113 out of 123 editing sites that are detectable in all six samples to correlate with the ~ 27,000 coding genes annotated in *A. thaliana*. Some sites have positive correlations with many genes while some other sites tend to show negative correlations or no correlation with gene expression (Fig. 3a). With multiple testing corrected [32] FDR < 0.05 in the Spearman correlation, we count the numbers of genes that are significantly correlated with editing levels of each site. Because the six samples contain three root samples and three shoot samples, it is reasonable to require the expression variation within three roots/three shoots to be smaller than the variation among six samples. Let SE = “standard error of mean”, based on the normal distribution of gene expression profile, we require SE_root_ < SE_all_ & SE_shoot_ < SE_all_ (root = three root samples; shoot = three shoot samples; all = six samples). Under these criteria, each editing site has less than 100 correlated genes (Fig. 3b). The 113 editing sites could be generally divided into three classes. Class I sites have more positively correlated genes, class III sites have more negatively correlated genes, and class II sites have very few genes correlated (Fig. 3b). Intuitively, it seems that class I and class III sites are more highly regulated than class II sites, so we guess that the class I and class III sites might be more essential or functional. Interestingly, class I and class III sites have significantly higher editing levels (Fig. 3c) and also less variable levels (Fig. 3d) than class II sites. Moreover, class I and class III sites have higher fractions of nonsynonymous (missense) editing sites than class II (Fig. 3e). Indeed, types I, II, and III sites are of different functions. Since the number of unique genes bearing editing events is too small to perform a gene ontology analysis, we would like to list the editing genes that are specific to type I, II, III sites. In other words, if a gene has both type I and type II sites, then this gene is excluded. In Table 1, we see that the genes specific to type I sites are chloroplast genes (gene ID beginning with ATCG, C stands for chloroplast) while the genes specific to type II and type III sites are mainly mitochondrial genes (gene ID beginning with ATMG, M stands for mitochondrial). This indicates the differential functions of the genes as well as the editing sites located on them.

**Table 1:** Genes specific to type I, II, and III editing sites

Type I/II/III site specific genes	Gene ID	Gene name
Type I site	ATCG00740	*RPOA* (RNA polymerase alpha subunit)
Type I site	ATCG00670	*CLPP1* (Caseinolytic protease P1)
Type I site	ATCG01050	*NDHD* (NAD(P) H dehydrogenase complex)
Type I site	ATCG00040	*MATK* (Maturase K)
Type II site	ATMG00180	*CCB452* (Cytochrome C biogenesis orf452)
Type II site	ATMG00690	*ORF240A* (FO-ATPase subunit)
Type II site	ATMG00080	*RPL16* (Mitochondrial ribosomal protein L16)
Type II site	ATMG00640	*ORF25* (Plant b subunit of mitochondrial ATP synthase)
Type II site	ATCG00180	*RPOC1* (RNA polymerase beta’ subunit-1)
Type II site	ATCG00300	*PSBZ* (Photosystem II subunit)
Type III site	ATMG01170	*ATP6–2* (ATPase subunit 6)

### Transcription factors show the strongest correlations with editing levels in different samples

The above paragraph focuses on the classification of editing sites according to their correlations with Ribo-seq gene expression levels. Here we wonder what kind of genes have the strongest correlation with levels of editing sites. We first define two sets of genes, n1 = genes with significant positive correlations with at least 10 editing sites, n2 = genes with significant negative correlations with at least 10 editing sites. Next, we define three classes of genes according to n1 and n2. The definition is as follows. “Positive” genes = set difference (n1 – n2) = n1 ⋂ ¬n2, “negative” genes = set difference (n2 – n1) = n2 ⋂ ¬n1, “both” genes = intersection (n1 & n2) = n1 ⋂ n2. With this definition, it is understandable that “positive” genes (331 genes) represent the genes with the greatest numbers of positively correlated editing sites, “negative” genes (479 genes) represent the genes with the greatest numbers of negatively correlated editing sites, and “both” genes (433 genes) have positive or negative correlations with different sets of editing sites.

**Fig. 4:**
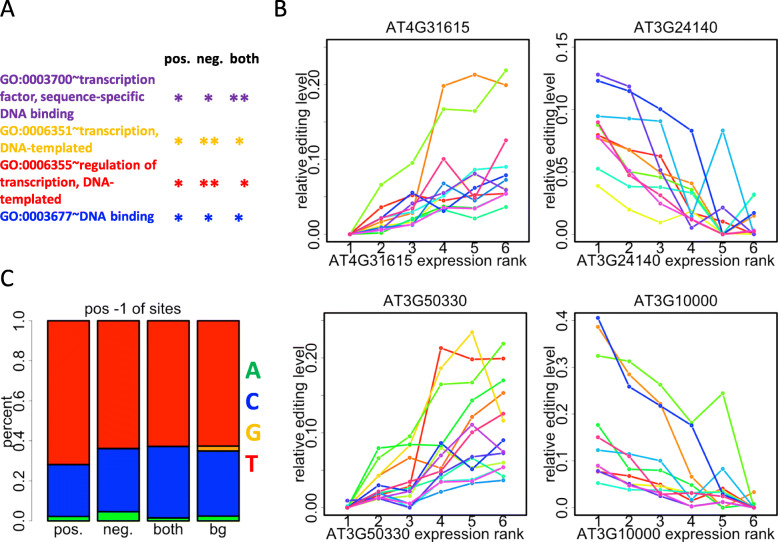
Transcription factors might play a regulatory role in affecting C-to-U editing levels. **a** GO (gene ontology) enrichment of three sets of genes. Genes are classified into three categories according to whether they contribute positively or negatively to the editing levels. *, FDR < 0.05; **, FDR < 0.01. **b** Two examples of transcription factors from positive genes and negative genes respectively. The X-axis represents six samples we use, and is ranked by increasing expression level (Ribo-seq RPKM) of the gene in each plot. Y-axis is the relative editing levels in six samples, and the lowest level among six samples is shifted to zero. Each line represents an editing site that show significant correlation with that gene. **c** Percentages of nucleotides at the 5-prime of editing sites. Editing sites are classified based on whether they are targeted by the “positive”, “negative” or “both” genes. “bg” represents the background composition of nucleotide at the 5-prime of the 123 C-to-U editing sites in our study

Interestingly, the functional annotation shows that all these three sets of genes are significantly enriched in transcription factors (Fig. 4a). We illustrate two examples of transcription factors from positive genes and negative genes respectively (Fig. 4b). The X-axis represents six samples we use, and is ranked by increasing expression level (Ribo-seq RPKM) of the gene in each plot. Y-axis is the relative editing levels in six samples, and the lowest level among six samples is shifted to zero. Each colorful line represents an editing site that show significant correlation with that gene (Fig. 4b). There is a clear trend that the two positive genes AT4G31615 and AT3G50330 contribute positively to the editing level while the two negative genes AT3G24140 and AT3G10000 show the opposite trend. AT4G31615 (REM35) is a transcriptional factor B3 family protein located in chloroplast, AT3G50330 is a bHLH transcription factor located in nucleus, AT3G24140 is a basic helix-loop-helix transcription factor located in nucleus, and AT4G10000 is located in chloroplast. The possibilities of how transcription factors affect the organelle editing would be discussed later.

As previous study proposed [33] or indicated from our data, different factors (genes) might be responsible for different editing sites. Based on this assumption, we pool the significantly correlated editing sites of positive, negative and both genes, respectively, and investigate the component of their 5-prime nucleotides (Fig. 4c). There is a higher fraction of thymidine at the 5-prime of editing sites (compared to background) which are correlated with positive genes (Fig. 4c). This result associates the trans-regulatory elements with the cis elements, suggesting that the editing sites controlled by different factors might have different sequence features.

At this moment, it is difficult to figure out how transcription factors could affect C-to-U RNA editing events, and there is no direct evidence to show that the trans determinants like transcription factors are related to the sequence motif around C-to-U editing sites. However, we stress the idea that the editing events might be indirectly affected by various factors apart from the PPR proteins. Moreover, the trans-regulatory elements and cis elements might be interconnected and it is not necessary to tear them apart in the functional studies as if they were independent.

## Discussion

Mutations act as the source for natural selection. Missense mutations lead to amino acid alterations and are intuitively subjected to selection force [34, 35]. Synonymous mutations do not change amino acids but they could be selected due to their impacts on mRNA splicing [36], codon optimality [37, 38], GC content [39], mRNA translation [40], and codon order [41]. Even the noncoding mutations could have a functional consequence by affecting microRNA targeting [42]. Since C-to-U RNA editing has similar consequences to mutations, these editing events must be carefully regulated by the organisms.

Apart from the PPR proteins as direct catalyzer of C-to-U conversion, other indirect trans or cis features may also contribute to the editing events. By analyzing the transcriptome and translatome data in *Arabidopsis thaliana* roots and shoots [26], combined with RNA-seq data from hybrids of *Arabidopsis thaliana* and *Arabidopsis lyrata* [28], we demonstrate that an upstream guanosine is unfavorable for the occurrence of C-to-U conversion. Surprisingly, this sequence context found for C-to-U editing sites is extremely similar to that of A-to-I RNA editing sites in animals [43–45], where a guanosine upstream of the edited adenosine is strongly avoided. In animals, the preference of sequence context around A-to-I editing sites is caused by the biochemical property of ADAR proteins [46]. Whether the context of C-to-U sites in plants is related to their catalytic enzyme remains to be investigated.

We also reveal that the double-stranded RNA regions are unfavorable for editing events. The structural basis we found could be supported by the fact that editing factors PPR proteins are likely to bind single-stranded RNAs [23]. More intriguingly, the animal A-to-I RNA editing events are also strongly affected by RNA secondary structures [47, 48] and the imperfectly-paired double-stranded RNAs are best substrates of ADAR. In plants, we found that C-to-U RNA editing sites are enriched in single-stranded (unstructured) RNA regions, which is opposite to the case of A-to-I editing. The structural basis for C-to-U or A-to-I RNA editing might not be a coincidence. If the editing events take place randomly in all regions without any specificity, then they are most likely to be non-adaptive and should be purged by natural selection. Note that the definition of double-stranded RNA is ambiguous for some loop regions in structured RNAs. It is debatable whether the loops belong to single- or double-stranded RNAs. Therefore, it is possible that the bonds in double-stranded RNAs prevent editing.

Meanwhile, by performing genome-wide correlation tests, we also find that different transcription factors might contribute positively or negatively to different editing sites. As we have mentioned, the previous study on mammalian A-to-I RNA editing [31] using GTEx mRNA-seq data has successfully found a new trans-regulatory elements AIMP2 that might affect the editing process. The cited literature also used correlation tests. Moreover, compared to mRNA-seq data, the Ribo-seq we use in our study might have stronger power to resemble the final protein amount. Indeed, transcription factors interact with DNA while the C-to-U editing events take place at RNA level. To discuss why nucleus genes could regulate the cytoplasmic editing, we raise some possibilities without further validation. First, the regulation is indirect. The transcription factors affect the gene expression of other related genes and those genes regulate RNA editing. Second, if the transcription factors indirectly regulate the expression level of editing genes, then there might be feedback loops controlling the editing status of those editing genes. Although the editing process does not take place in nucleus, the transcription factors might change editing levels via controlling the amount of editing transcripts. At this moment, it is hard to attribute the editing level fluctuations to the transcription factors. The molecular mechanisms remain to be investigated.

Last but not least, it is hypothesized that C-to-U RNA editing in plants could be designed for reversing the potentially unfavorable T-to-C DNA mutations [49] and therefore the editing levels should be constantly high to mimic the DNA mutation. Our results show that although the editing levels are fluctuating across samples, the range of the most variable levels is usually less than 20% as shown in Fig. 4b. Thus, our study does not conflict with established knowledges. We add new aspects in depicting the C-to-U RNA editing mechanisms in plants and would be appealing to the broad plant biologists.

## Conclusions

Our study reveals that the 5-prime nucleotide and the RNA secondary structures affect C-to-U RNA editing in cis. An upstream guanosine or double-stranded RNA regions are unfavorable for editing events. Meanwhile, many genes including the transcription factors play regulatory roles in trans. Different transcription factors might contribute positively or negatively to different editing sites.

## Methods

### Data collection

The reference genome and CDS sequences of *Arabidopsis thaliana* were downloaded from TAIR database. The TAIR 9 version of annotation was used. The mRNA-seq and Ribo-seq data of *Arabidopsis thaliana* (three replicates for roots and shoots) were retrieved from a previous study [26]. As described by the paper, the *Arabidopsis* Columbia-0 (abbr. Col-0) seeds (around 1500 seeds per vessel) were surface sterilized and imbibed at 4 °C for 2 days. They were grown hydroponically on fine nylon mesh supported by a customized rack in Magenta vessel GA-7-3 (Sigma; V8380) with filtered sterile liquid media and shaken at 85 rpm under 16-h light (110–115 μmol m^− 2^·s^− 1^ from cool white fluorescent bulbs) and 8-h dark at 22 °C [26]. The tissues were collected from 4-day old seedlings.

The RNA-seq data from hybrids of *Arabidopsis thaliana* and *Arabidopsis lyrata* were generated by a previous study [28] under accession number SRP073606 (100 bp, pair-ended). In the cross experiment, *A. thaliana* was used as a mother (Col-0, obtained from the Arabidopsis Biological Resource Center, ABRC, USA) and *A. lyrata* was used as a father (*Arabidopsis lyrata ssp. lyrata* genotype MN47). Hybrid seeds were germinated and grown on germination medium containing Murashige and Skoog salts, 1% sucrose, and 0.8% agar. The plants were stratified for 5 days at 4 °C, and then grown for 4 weeks in a growth chamber at 22 °C /16 °C under 16 h light/8 h dark. Then, different treatments were done to the hybrid samples. For dehydration, plants were removed from the agar and dehydrated in plastic dishes for 1 h at 22 °C under dim light (0.7 ± 0.8 mmol s^− 1^ m^− 1^). For cold exposure, plants were grown under dim light (0.7 ± 0.8 mmol s^− 1^ m^− 1^) at 4 °C for 1 h. Leaf samples of plants growing in non-stressful conditions (standard treatment) were collected on 4 week-old plants grown at 22 °C. To control for circadian changes in gene expression, all samples were collected at 12:00 pm. In the mRNA-seq data analysis, to increase the power in detecting RNA editing sites, all the mRNA-seq libraries from different conditions including the control samples are pooled.

### Mapping the NGS reads

For the *A. thaliana* data, we map the mRNA-seq reads to the reference CDS sequences using Bowtie2 [30]. For the hybrids of *A. thaliana* and *A. lyrata*, we only focus on the C-to-U editing sites detected in chloroplast and mitochondrial genes.

The *A. lyrata* genome (CDS sequences) is downloaded from the Ensembl website with the following code (wget ftp://ftp.ensemblgenomes.org/pub/plants/release-44/fasta/arabidopsis_lyrata/cds/Arabidopsis_lyrata.v.1.0.cds.all.fa.gz). We combine the chloroplast and mitochondrial genomes of these two species and map the hybrid mRNA-seq data to the combined genome with Bowtie2. We extract uniquely mapped reads so that only the regions that are non-identical between *A. thaliana* and *A. lyrata* could be covered (otherwise the read would be mapped at least twice). The uniquely mapped reads are extracted using SAMtools [50].

### Variant calling

The variant calling process is accomplished by SAMtools “mpileup”. The minimum mapping quality of reads is set to be 20 (parameter –q 20) and minimum base quality is set to be 30 (parameter –Q 30) to increase the accuracy of variation sites. The output file of mpileup is “vcf” format, which contains one variation site per line. The information for each variation site includes total depth on each site, the reference allele count and each alternative allele count. Sites with more than one variation type are discarded. Variation level = alternative allele count / total depth. Take C-to-U RNA editing sites for instance, editing level = T/(C + T). To avoid the false positive variants caused by technical limitations, the variant sites with levels higher than 0.05 and with at least 10 covered reads are maintained [51–53]. Note that one gene could have multiple isoforms so that some variation sites appearing in different isoform might belong to the same genomic location. Thus, only the longest CDS was used for variant calling to avoid the redundancy.

In the RNA-seq of hybrid, with the uniquely mapped reads, we have verified two species-specific sites with adequate sequencing depth. Two cytidines, position 362 and position 376 on gene ATMG00580 (NADH dehydrogenase subunit 4), are fully edited in *A. thaliana* (103 reads covered) and completely unedited in *A. lyrata* (31 reads covered).

### RNA structure

We use RNALfold [54] to determine the regions with RNA secondary structures. Each CDS sequence is input to the software. With default parameters, the output file would contain the positions of all the secondary structures within the input sequence. We merge the structured regions within each CDS. The length of structured regions divided by the length of each CDS is the structure% for each gene. Our loose criterion considers all the structured regions with Z-score < 0 reported by the software. All the CDSs we used contain 33.2 million bases and all the structured regions under our criterion contain 18.4 million bases (18.4/33.2 = 55%). Among the 123 C-to-U editing sites we identified, 43 of them are located in structured regions (43/123 = 35%).

The diagram of RNA structure is accomplished with online tools RNA structure (http://rna.urmc.rochester.edu/RNAstructureWeb/Servers/Predict1/Predict1.html). The only input message needed is the nucleotide sequence.

### Gene expression analysis

The expression level of a gene is defined as RPKM (reads per kilobase per million mapped reads). In the correlation test between editing levels and gene expression, the RPKM of Ribo-seq reads are used to represent the expression of a gene.

### Functional annotation

The functional annotation of the genes IDs is performed using the online software DAVID [55].

### Statistical analysis and code availability

All statistical analyses (for example, correlation tests) and the graphic work were conducted in R environment (http://www.R-project.org/). All codes used in the analyses are available under request.

## Data Availability

All data used in this study are public data, the sources or links of which are provided in the Materials and Methods.

## Abbreviations

mRNA: Messenger RNA
CDS: Coding sequence
NGS: Next generation sequencing
nsy: Nonsynonymous
syn: Synonymous
